# A Fluorometric Assay for the *In Vitro* Evaluation of Activity against Naegleria fowleri Cysts

**DOI:** 10.1128/spectrum.00515-22

**Published:** 2022-07-11

**Authors:** Iñigo Arberas-Jiménez, Aitor Rizo-Liendo, Ines Sifaoui, Javier Chao-Pellicer, José E. Piñero, Jacob Lorenzo-Morales

**Affiliations:** a Instituto Universitario de Enfermedades Tropicales y Salud Pública de Canarias, Universidad de La Laguna, La Laguna, Spain; b Departamento de Obstetricia, Ginecología, Pediatría, Medicina Preventiva y Salud Pública, Toxicología, Medicina Legal y Forense y Parasitología, Universidad de La Laguna, La Laguna, Spain; c Red de Investigación Colaborativa en Enfermedades Tropicales (RICET), Madrid, Spain; d Consorcio Centro de Investigacion Biomedica En Red M.P. (CIBER) de Enfermedades Infecciosas (CIBERINFEC), Instituto de Salud Carlos III, Madrid, Spain; Barcelona Centre for International Health Research (CRESIB, Hospital Clinic-Universitat de Barcelona)

**Keywords:** antiamoebic activity, cyst, Naegleria, meningoencephalitis, protocol

## Abstract

Primary amoebic meningoencephalitis (PAM) is a lethal and rapid infection that affects the central nervous system and is caused by the free-living amoeba Naegleria fowleri. The life cycle of this protozoa consists of three different stages: The trophozoite, flagellate and cyst stages. Currently, no fully effective molecules have been found to treat PAM. In the search of new antiamoebic molecules, most of the efforts have focused on the trophozoidal activity of the compounds. However, there are no reports on the effect of the compounds on the N. fowleri cyst viability. In the present study, the cysticidal activity of four different molecules was evaluated using an alamarBlue based fluorometric assay. All the tested compounds were active against the cyst stage of N. fowleri. In fact, all the molecules except the amphotericin B, showed highest activity toward the cyst stage than the trophozoite stage. This work could be an effective protocol to select molecules with cysticidal and trophozoidal activity that can be considered a future PAM treatment.

**IMPORTANCE** In the search of new anti-Naegleria fowleri compounds, most of the works focus on the activity of different molecules against the trophozoite stage; however, none of them include the effect of those compounds on the cyst viability. This manuscript presents a solid and reliable assay to evaluate the activity of compounds against the cyst stage of N. fowleri.

## INTRODUCTION

Naegleria fowleri is the causative agent of the primary amoebic meningoencephalitis (PAM), a fatal disease in which more than the 95% of the reported cases affects the central nervous system of children and young adults (1, 2). The infection occurs when the trophozoite enters the nasal cavity of the patient after performing risky activities in untreated water sources. Initial symptoms, which include intense headache, temperature, stiff neck, and seizures (3, 4) appear during the first 9 days after the exposure to the contaminated water source whereas the average time of patient's death is between 1 and 18 days after the first symptoms appearance (5, 6). Current therapy options involve a combination of different drugs, including amphotericin B, miltefosine, azithromycin, rifampin, and azoles (7, 8).

The life cycle of this protozoon consists of three different stages depending on environmental conditions (9): the trophozoite, flagellate, and cyst stages (cystSt). The ameboid trophozoites measure from 10 to 25 μm and reproduce by binary fission. The trophozoites can turn into flagellate stage in aqueous environments with lack of nutrients (10, 11) or when the ionic concentration of the media changes (12). The cystSt of N. fowleri can be found under unfavorable conditions, namely high temperatures, low pH-environments, or high salinity levels (13).

On the other hand, and despite that water is the most frequent source of infection, the inhalation of cyst-laden dust and the subsequent excystation has also been proposed as a route of infection (6, 14). Even though the presence of cysts in brain of PAM patients has not been reported (15), the possibility of an encystation process of trophozoites after the treatment administration and, hence, the risk of recurrent infections should not be excluded.

Prior to this current study, research in this area have focused on evaluating the drug effect on the trophozoite stage (3). However, we could find no report using the rapid fluorescence method to measure the effect of drugs on the N. fowleri cyst viability.

In this work, the anticyst activity of two reference drugs, amphotericin B and miltefosine, and two algae isolated molecules with proved antitrophozoite activity (16, 17), staurosporine and laurinterol, was evaluated.

## RESULTS

As shown in Table 1, staurosporine was the most active molecule against both trophozoites and cysts with similar values for both stages, 0.08 ± 0.01 μM (16) and 0.06 ± 0.01 μM, respectively. Regarding the other natural compound, laurinterol, the results showed also similar antitrophozoite and anticyst IC_50_s with 13.42 ± 2.57 μM (17) and 8.81 ± 1.08 μM values, respectively.

**TABLE 1:** Inhibitory concentrations 50 (IC_50_) of evaluated compounds against cyst and trophozoite stage of Naegleria fowleri ATCC 30808 strain^*a*^

Compound	IC_50_ (μM) against cyst stage	IC_50_ (μM) against trophozoite stage
Staurosporine	0.06 ± 0.01	0.08 ± 0.01
Laurinterol	8.81 ± 1.08	13.42 ± 2.57
Amphotericin b	0.53 ± 0.03	0.12 ± 0.03
Miltefosine	21.52 ± 2.62	38.74 ± 4.23

a Experiments were made in triplicate in three different and independent assays and the mean values and standard deviation were also calculated.

On the other hand, both natural molecules were more active than the reference drug miltefosine which showed an IC_50_ of 21.52 ± 2.62 μM against the cysts and 38.74 ± 4.23 μM against the trophozoites (18). Finally, amphotericin B showed similar values to staurosporine. However, this was the only compound with a higher IC_50_ value against the cystSt (0.53 ± 0.03 μM) than the obtained for the trophozoite stage (0.12 ± 0.03 μM) (18).

Additionally, the ability to inhibit the excystation in the presence of the compounds was also checked. Trophozoites could be seen 24 h after incubation with staurosporine at 0.26 μM, laurinterol at 10.58 μM, amphotericin B at 0.33 μM, and miltefosine at 36.80 μM.

## DISCUSSION

In the present study, the anticyst activity against N. fowleri of four molecules is described. As seen in Table 1, the anti-*Naegleria* activity was similar against the cyst and trophozoite stages in all the tested compounds except in the case of the amphotericin B. Moreover, this compound was the only one in which the anticyst activity is lower than the antitrophozoite one. The biochemical mechanism of the amphotericin B in N. fowleri is based on the lysis of the cell membrane and pore formation by binding to ergosterol and, hence, altering the membrane permeability (19, 20). In the cystSt of N. fowleri, the cytoplasm is protected by a cyst wall whose composition is not clear (21); however, Chávez-Munguía et al. reported the presence of enolase in the cyst wall (22). Enolase has been proposed as cholesteryl ester hydrolase inhibitor, an enzyme that catalyzes the hydrolysis of the cholesteryl ester to obtain cholesterol (23). Moreover, a “switch” between different types of sterols have been hypostatized during *Naegleria*, e.g., encystation/excystation processes (24). Taking this into account, the difference in the activity shown by amphotericin B between the trophozoite and cysts stages could be explained by the loss of the sterol quantity present in the cysts wall compared with the trophozoite’s cellular membrane.

**FIG 1:**
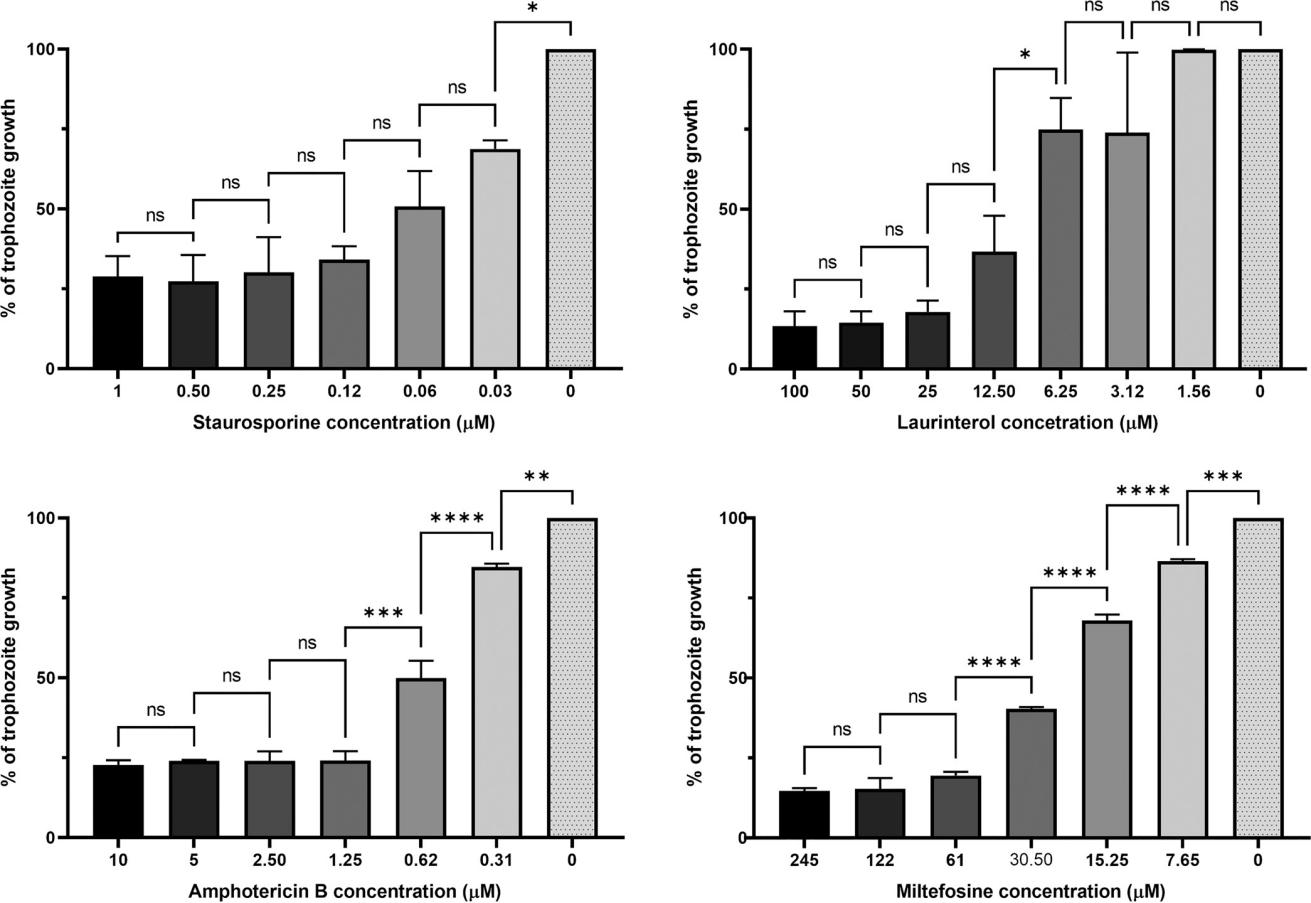
Dose-response assessment for each drug against N. fowleri cysts after 96 h. Bars represent the trophozoite growth (estimated by the resazurin reduction) after the excystation, thus the percentage of cysts that remain viable after the treatment is measured. Experiments were made in triplicate in three different and independent assays and the mean values and standard deviations were also calculated. Differences between the values were assessed using one-way analysis of variance (ANOVA). Data are presented as means ± SD. NS, not significant; *, *P* value < 0.05; **, *P* value < 0.01; ***, *P* value < 0.001; ****, *P* value <0.0001.

Furthermore, all the tested molecules showed cysticidal activity (Fig. 1), which together with the antitrophozoite activity make them more complete and effective molecules for the PAM treatment. In particular, those two natural compounds obtained from the marine environment constitute promising candidates to be considered for the future PAM treatment not only because of their anti-*Naegleria* properties, but also taking into account their low cytotoxicity. In previous works, the cytotoxic concentration 50 (CC_50_) of the staurosporine and laurinterol was reported being 8.74 ± 0.72 μM (16) and 80.11 ± 7.79 μM (17), respectively. Moreover, the mechanism of action that induce both molecules in treated amoebae was also described (16, 17), showing a programmed cell death. In fact, cell death induction by a necrotic process can cause the presence of undesired effects, while the programmed cell death avoids the inflammation in treated patients and leads to a safer therapy (25). However, more studies are needed to confirm the efficacy and toxicity of the compounds in live organisms.

**FIG 2:**
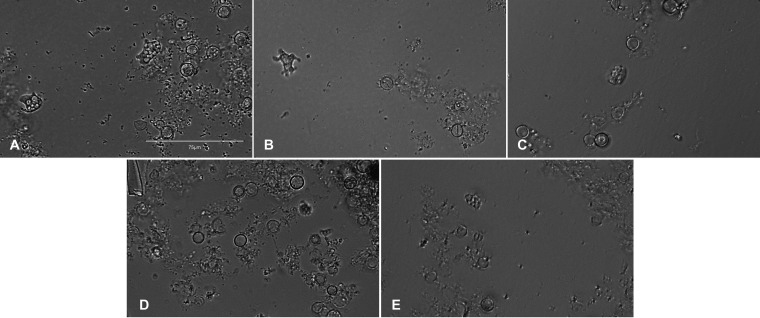
N. fowleri cysts after 24 h of incubation with the evaluated compounds. Staurosporine (B) at 0.26 μM, laurinterol at 10.58 μM (C), amphotericin B (D) at 1.35 μM and miltefosine (E) at 36.80 μM. Negative control (A) consisted of the cysts with bactocasitone. Images (40 ×) were obtained using an EVOS M5000 Cell Imaging System, Life Technologies, Spain. Scale bar, 75 μm.

Furthermore, this work represents the first attempt to evaluate the anticyst activity *in vitro* using the alamarBlue reagent which could be an effective protocol to select molecules exhibiting cysticidal and trophocidal activities. In fact, the colorimetric assay based on the alamarBlue reagent is a well-established protocol which is widely used in the search of new antiamoebic compounds against the trophozoite stage of N. fowleri (26). Moreover, the IC_50_ values of the reference drugs, amphotericin B and miltefosine, were also evaluated using the CellTiter-Glo luminescent assay, showing similar results (IC_50_ = 0.09 ± 0.03 for amphotericin B; IC_50_ = 54.5 ± 0.01 μM for miltefosine) (27) to the ones obtained by our group. On the one hand, this assay enables the evaluation of the potential of the compounds to inhibit the excystation (while the compounds are incubated with the cysts) (Fig. 2). In addition, the viability of the remaining cysts is also tested as the medium is changed to fresh bactocasitone medium allowing the excystation. Finally, using the alamarBlue colorimetric assay (18) and the newly excysted trophozoites, the inhibitory concentration 50 (IC_50_) can be measured.

## MATERIALS AND METHODS

### Chemicals.

Amphotericin B was purchased from Sigma-Aldrich (Madrid, Spain). Miltefosine was acquired from Cayman Chemicals (Vitro SA, Madrid, Spain). Staurosporine was isolated from the Streptomyces sanyensis PBLC04 strain (collected in Ecuador) (16). Laurinterol was obtained from the *Laurencia johnstonii* red algae, which was collected in Baja California, Mexico (17). Stock solutions of the compounds were prepared in dimethyl sulfoxide (DMSO) and maintained at −20°C until required.

### Cell cultures.

A type strain of N. fowleri (ATCC 30808) was used. Trophozoites were harvested and transferred from Bactocasitone medium to MYAS medium which is composed by: malt extract (Difco B 186), 0.1 g; yeast extract (Difco B 127), 0.1 g; and 1 L of amoebae saline according to the instructions of the Culture Centre of Algae and Protozoa. Composition of amoebae saline: NaCl, 0.12 g; MgSO4.7aq, 0.004 g; CaCl2.2aq, 0.004 g; Na2HPO4, 0.142 g; KH2PO4, 0.136 g; distilled water, 1 L (28). The cells were maintained in this medium with slight agitation for 10 days in order to obtain mature cysts.

### *In vitro* activity assays against N. fowleri cysts.

Serial dilutions of the evaluated compounds in Bactocasitone medium were made on a 96-microtiter plate (Thermo Fisher Scientific, Madrid, Spain). On the other hand, 0.5% of SDS was added to the mature cyst culture to eliminate the nonviable cells. After 10 min, SDS treated cells were centrifuged and resuspended in bactocasitone medium to obtain a 2 × 10^5^ cysts/mL stock solution. Finally, 50 μL of the stock solution of the cysts were added to the 96-microtiter plate. For the negative control, the cysts with the medium alone were seeded. Plates were incubated in slight agitation for 24 h at 37°C. The final volume was 100 μL in each well.

After that, plates were centrifuged, and the supernatant was removed and replaced with 100 μL of fresh Bactocasitone medium in order to ease the excystation. Finally, 10 μL of the alamarBlue Reagent was placed into each well and the plates were incubated for 72 h at 37°C.

Plates were analyzed with the EnSpire Multimode Plate Reader (PerkinElmer, Madrid, Spain) at 570/585 nm. The percentages of growth inhibition 50% (IC50) were calculated performing a nonlinear regression analysis with a 95% confidence limit using the GraphPad Prism8.0.2 software program (GraphPad Software, San Diego, CA, USA). All activity assays were made in triplicate in three different and independent experiments and the mean values were also calculated. A one-way analysis of variance (ANOVA) was used for the analysis of the data and the values of *P* < 0.05 were considered statistically significant.

The alamarBlue colorimetric assay is based on the reduction of the nonfluorescent resazurin to the fluorescent resorufin in the presence of metabolic activity. Thus, the reduction of the resazurin and consequently the fluorescence, is produced by the trophozoites that emerge from the cysts.
